# Outbreak of *Escherichia coli* O157 associated with pork products linked to colonized swine in Alberta, 2018

**DOI:** 10.14745/ccdr.v52i09a03

**Published:** 2026-09-03

**Authors:** Lance Honish, Julia Keenliside, Saida Essendoubi, Linda Chui, Nyall Hislop, Sarah Stephen, Kristin Hill, Christine Kopko, Gary Gensler, Victor Mah, Tanis Kershaw

**Affiliations:** 1 Alberta Health Services, Edmonton, AB; 2 Julia Keenliside Consulting, Edmonton, AB; 3 Alberta Ministry of Agriculture and Irrigation, Edmonton, AB; 4 Alberta Precision Laboratories, Public Health Laboratory (ProvLab), Edmonton, AB; 5 University of Alberta, Edmonton, AB; 6 Canadian Food Inspection Agency, Ottawa, ON; 7 Alberta Ministry of Primary and Preventative Health Services, Edmonton, AB; 8 Centre for Food-borne, Environmental & Zoonotic Infectious Diseases, Public Health Agency of Canada, Guelph, ON

**Keywords:** *E. coli* O157, outbreak, pork, swine, foodborne illness

## Abstract

**Background:**

In March 2018, an outbreak of *Escherichia coli* (*E. coli*) O157 infections was identified in Alberta. The objective of this article is to describe the outbreak, including the epidemiological, food safety and laboratory investigations that contributed to the identification of the outbreak source.

**Methods:**

Outbreak cases were identified through notifiable disease surveillance. Exposure (including food consumption) information was collected from outbreak cases. Onsite investigations were conducted at food facilities linked to outbreak case exposures, including collection of environmental swabs and food samples for *E. coli* O157 analysis and traceback investigation.

**Results:**

There were 43 outbreak cases identified; 33% of cases were hospitalized and one case was fatal. Dates of illness onset ranged between March and May 2018. An *E. coli* O157 outbreak strain was identified in outbreak case isolates and in 20 samples collected as part of this investigation (pork, swine feces and environmental swab). Implicated pork was traced back to a provincially licensed abattoir and meat processing facility. Outbreak cases were linked to six food facilities; 51% of cases were linked to one restaurant facility. Interventions included recall of implicated pork products, and temporary closure and implementation of food handling process improvements at implicated facilities.

**Conclusion:**

This investigation demonstrates a direct link between colonized swine, contaminated pork products and human *E. coli* O157 illnesses. The findings highlight the importance of consumer guidance for safe pork handling, as well as interventions during animal husbandry, biosecurity, slaughter, processing and handling to reduce the risk of human *E. coli* O157 illnesses.

## Introduction

Shiga-toxin-producing *Escherichia coli* (STEC), including *E. coli* O157, is a major cause of foodborne illness in humans and is associated with morbidity and mortality worldwide. In 2019, there were 397 laboratory-confirmed *E. coli* O157 illnesses in Canada, a rate of 1.06 illnesses per 100,000 population; in Alberta, the rate was 2.17 per 100,000 population (1). In Canada, approximately 60% of *E. coli* O157 illnesses have been attributed to foodborne transmission (2). Furthermore, it is estimated that beef and raw produce are the source of 47.4% and 19.5% of foodborne *E. coli* O157 illnesses, respectively; for pork, the estimate is 2.0% (3). Pork-associated *E. coli* O157 outbreaks are relatively uncommon in Canada (4); in Alberta, however, there have been two previously reported *E. coli* O157 outbreaks attributed to pork products in 2014 (5) and 2016 (6). Between March and May 2018, an outbreak of 43 *E. coli* O157 infections associated with pork products was investigated in Alberta, Canada. The objective of this article is to describe the outbreak, including the epidemiological, food safety and laboratory investigations that contributed to the identification of pork products from infected swine as the source, describe the public health interventions implemented to prevent further illnesses, and highlight that pork should be considered as a vehicle of transmission of *E. coli* O157 infections.

## Methods

### Overview and outbreak detection

Shiga-toxin-producing *E. coli* is a notifiable disease in Alberta and the rest of Canada. The outbreak investigation began on March 27, 2018, when Alberta Health Services (AHS) identified a sudden increase above baseline incidence of *E. coli* O157 cases in one AHS Zone through notifiable disease surveillance, with two cases reporting consumption of food from the same restaurant. The outbreak was declared over on June 1, 2018, following two maximum *E. coli* O157 incubation periods (20 days) without a new case linked to the outbreak.

### Outbreak case finding and data collection

The case definition for the outbreak was as follows: a resident of, or visitor to, Alberta with laboratory-confirmed *E. coli* O157 infection matching the outbreak pulsed-field gel electrophoresis (PFGE) pattern combination, and illness onset on or after March 1, 2018. Outbreak cases were identified through notifiable disease surveillance and subsequent PFGE characterization of case isolates. Individuals meeting the case definition for *E. coli* O157 were interviewed using a standard AHS public health follow-up questionnaire to collect relevant clinical, demographic and exposure information. When this approach did not reveal the outbreak source, open-ended re-interviews were conducted, including face-to-face discussion in case homes and at area hospitals.

### Epidemiologic and statistical analysis

Frequency of outbreak case exposures from completed questionnaires were analyzed, including consumption of specific foods and purchase of food from specific food facilities. Microsoft Excel was used to analyze the data, generate descriptive statistics and create charts. An epidemiological study (e.g., case-control) was not conducted; case series analysis yielded compelling source attribution data.

### Food safety investigation

The outbreak was investigated by member agencies of the Canada-Alberta Partners in Food Safety Alberta Foodborne Illness and Risk Investigation Protocol (FIRIP) (7) after initial case investigation revealed illnesses were likely foodborne. The FIRIP partner agencies conducted inspections and onsite investigations at implicated food facilities. They collected environmental swabs and food samples from those facilities, as well as food samples from outbreak case homes. Employees of an implicated restaurant submitted clinical specimens for analysis, as required by the local Medical Officer of Health (MOH). Swine and cattle fecal samples were also collected from the implicated abattoir, meat processing facility and live animal production areas. Samples were submitted to Alberta Precision Laboratories, Public Health Laboratory (ProvLab), Alberta Agriculture and Irrigation (AGI; formerly Alberta and Forestry), and the Canadian Food Inspection Agency (CFIA) laboratories for *E. coli* O157 isolation and characterization.

### Laboratory investigation

Environmental and food samples were analyzed following methods published in the Health Canada Compendium of Analytical Methods (8), namely, MFLP-30 and/or MFHPB-10 (with and without modifications), as well as MFLP-22, in some cases. All *E. coli* O157 isolates (clinical, food and environmental) were characterized by PFGE and subsequently by whole-genome multilocus sequence typing (wgMLST) using the standardized PulseNet International Protocol. For PFGE, all isolates were digested by *Xba*I and *Bln*I restriction enzymes followed by analysis using BioNumerics 6.01 and a comparison of representative PFGE profiles was prepared using the Dice coefficient and Unweighted Pair Group Method with Arithmetic Mean (UPGMA) cluster analysis. Pulsed-field gel electrophoresis patterns were uploaded to the PulseNet Canada (PNC) database located at the Public Health Agency of Canada National Microbiology Laboratory (NML), Winnipeg; a national pattern was designated. As for wgMLST, the analysis was performed using BioNumerics 7.6.

### Public health interventions

Public health interventions initiated to control the outbreak included:

• Recall of raw pork products possibly contaminated with *E. coli* O157

• Exclusion of *E. coli* O157-infected employees working at an implicated restaurant identified through MOH-required screening and voluntary closure of the restaurant pending screening results

• Implementation of food handling process improvements at implicated facilities, under the supervision of FIRIP partners

• Public notification via news releases regarding the outbreak and *E. coli* O157 prevention measures

## Results

There were 43 outbreak cases identified. Outbreak case mean and median ages were 27 and 23 years, with a range of 1–85; 16 cases (37%) were aged six years or less. Twenty-five (58%) cases were male. Predominant symptoms reported were diarrhea (97%), bloody stool (89%), and abdominal pain (54%). Fourteen (33%) cases were hospitalized, and five (12%) developed hemolytic uremic syndrome. One case was fatal. Four (9%) were secondary cases (i.e., household contact of another outbreak case). Most cases resided in one metro region of Alberta (35, 81%); six cases resided elsewhere in Alberta, and two cases were non-Alberta residents. Dates of illness onset or specimen collection dates ranged between March and May 2018 (**Figure 1**). Mean incubation period was 3.3 days with a range of 2–5 days (among cases with a known suspected exposure date).

**Figure 1:**
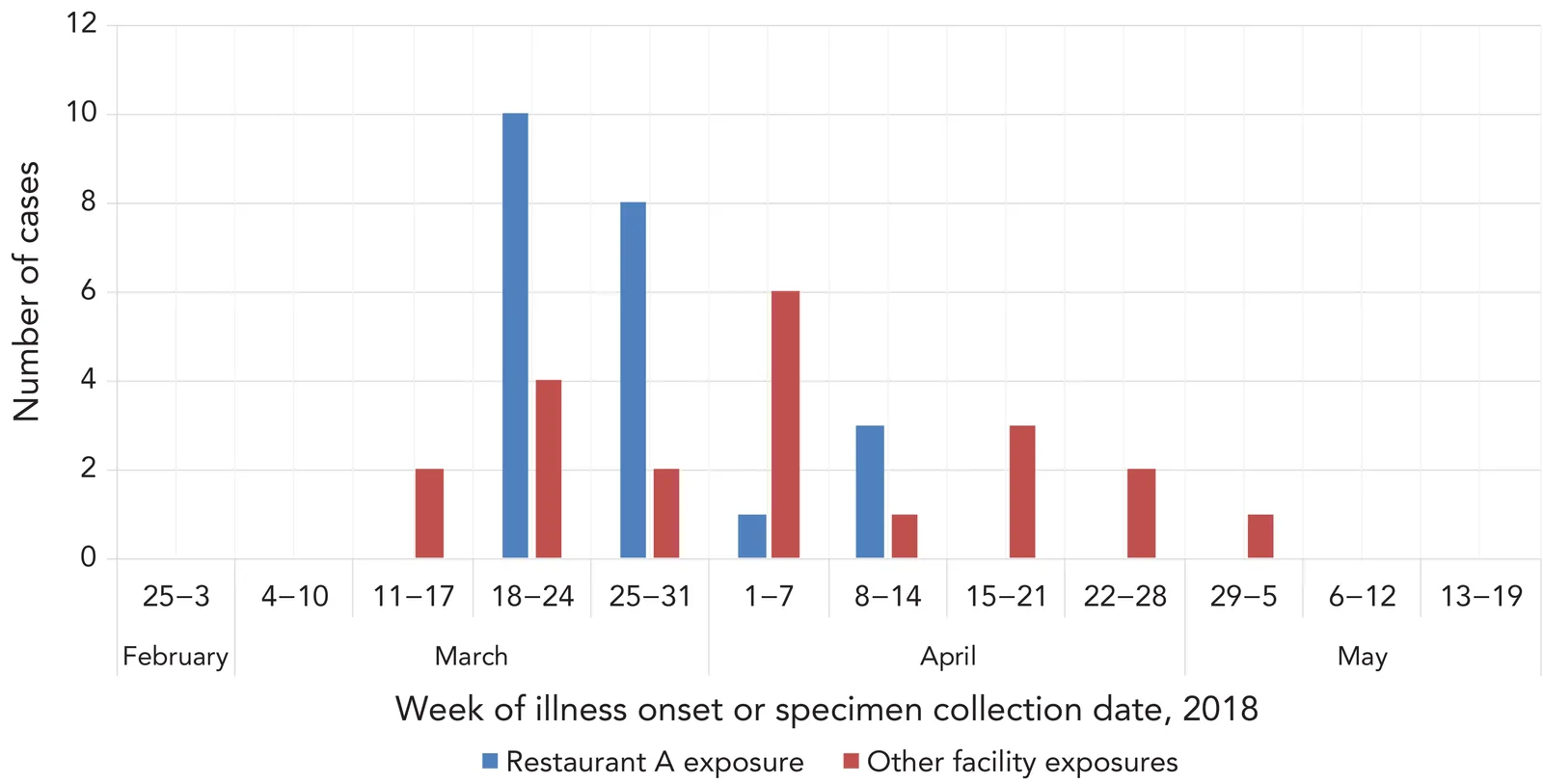
Case distribution by week of illness onset or specimen collection date and facility exposures, pork-associated outbreak of *Escherichia coli* O157, Alberta, March–May 2018 Note: The epidemiologic curve shows the number of outbreak cases illustrated by month and week of symptom onset or specimen collection date. Cases occurred between March and May of 2018. During the weeks of March 11–April 29, 2018, there were 1 to 14 cases. There were two cases during the week of March 11, and in the two-week period March 18 to 31, there was a peak of 24 outbreak cases. The number of weekly cases steadily declined in the remaining weeks of the outbreak. Outbreak cases are also illustrated by facility exposures. Cases with exposure to Restaurant A occurred between the weeks of March 18 to April 8, with 1 to 10 cases reported each week. Cases with other facility exposures took place between the weeks of March 11–April 29, 2018, with 1 to 6 cases reported each week

Case exposure investigation and traceback revealed that 35/43 cases had direct or indirect exposure to food from a provincially licensed meat facility (Facility A) via an Alberta restaurant, Restaurant A (22/35), and other facilities in Alberta that processed pork from Facility A (13/35, **Table 1**). Seven cases consumed pork products purchased from Retail Establishment A, four of whom consumed mettwurst, a sausage product understood to contain raw pork not cured or smoked and sold as ready-to-eat. Production records revealed that during the timeframe of interest, Facility A supplied pork to Retail Establishment A. Further investigation identified four additional facilities (Retail Establishment B, Retail Establishment C, Retail Establishment D, Restaurant B) that produced pork products consumed by outbreak cases, which received pork from Facility A, directly or through secondary points of sale. Eight cases had indeterminate or no known exposure to pork originating from Facility A.

**Table 1:** Case exposures to facilities that received pork from Facility A, pork-associated outbreak of *Escherichia coli* O157, Alberta, March–May 2018

Facility	Number exposed^a^	Reported pork consumption (cooked, raw/undercooked)	Facility employee	Secondary case
Restaurant A	22	7^b^ (7, 0)	7	3
Retail Establishment A	7	7 (3, 4^c^)	N/A	N/A
Other facilities^d^	6	5 (4, 1^e^)	N/A	1

Abbreviation: N/A, not applicable

^a^ Not shown: six cases with indeterminate exposure, two cases with no known exposure to pork originating from Facility A

^b^ Among 12 restaurant patron cases

^c^ Consumed ready-to-eat sausage product containing raw pork

^d^ Includes Retail Establishment B, Retail Establishment C, Retail Establishment D, Restaurant B

^e^ Consumed pork intentionally undercooked

### Food safety investigation—Restaurant A

Twelve cases reported consuming food from Restaurant A as direct or indirect patrons of the restaurant. Meal consumption dates ranged between March 16 to March 24. Seven cases reported consumption of pork in the restaurant meal. Complete and detailed food histories were challenging to collect: six cases reported consumption of food served in the “Boodle” style (assorted cooked meats, rice and vegetables served on banana leaves and eaten by group dining parties (9)), two cases consumed leftover food provided by restaurant patrons and two cases were vague historians. Three cases that reported no consumption of food from Restaurant A were household contacts of Restaurant A patrons that developed diarrhea within seven days of the meal but were not tested for *E. coli* O157.

Investigators visited the restaurant on and between March 27 and April 18. During inspections, the following were noted: several containers of meats kept at room temperature, unsanitary conditions in food preparation areas, and evidence of a rodent infestation in the facility. Violations were corrected and included completion of an online food safety course by restaurant employees.

Restaurant A staff (18 in total) submitted stool specimens for *E. coli* O157 screening as required by the MOH; specimens from seven employees were *E. coli* O157 culture positive (see Table 1). All employees identified as *E. coli* O157-infected via stool screening worked at the restaurant during the implicated meal date period. Six cases reported having no diarrhea at any time between March 1 and the date their specimen was collected; one employee reported onset of diarrhea on March 31 that lasted one day. Ready-to-eat food handled by infected employees was discarded upon request. Infected employees were excluded by MOH order from food handling occupations pending submission of two consecutive *E. coli* O157-negative stool specimens. The restaurant voluntarily closed on April 2 and reopened on April 18.

### Food safety investigation—Facility A

Facility A is a provincially licensed abattoir located in Alberta that slaughters swine and processes various beef and pork products for retail, restaurants and direct sale. This facility also includes a 100-sow farrow to finish swine barn, and a 1,500-cattle feedlot operation on the same premises in close proximity to the abattoir. Swine slaughtered and processed in Facility A were all from the premises. Beef cattle from the premises were slaughtered off-site at a federally licensed plant, and some beef product was brought back and processed on site.

Alberta Agriculture and Irrigation staff conducted root cause analyses in the meat processing area at Facility A that revealed three general areas for improvement: a lack of record-keeping to verify process controls, inadequate handling of the ready-to-eat product, and inadequate slaughter procedures. Further investigation demonstrated that the operators and staff needed further training and explanation on how pathogens can enter the food chain during slaughter, dressing, and processing. The operator and staff were very responsive to AGI’s recommendations.

On April 18, at AGI request, all products at Facility A were held pending further investigation. On April 24, Facility A voluntarily recalled all raw pork products produced at the facility on or between February 19 and April 24. This resulted in over 100 recalled pork products produced or sold by eight facilities, as communicated in CFIA recall notices on or between April 24 and May 2. Foodborne Illness and Risk Investigation Protocol partners collaborated on recall effectiveness checks for the affected products.

Alberta Agriculture and Irrigation conducted further investigation at Facility A in May 2018. The swine barn was located within a few hundred metres of the cattle feedlot. The cattle in the feedlot were sourced directly from a small number of farms. No illness was reported or observed in either the swine or the cattle during the outbreak period, although *E. coli* O157 infection in these species is typically asymptomatic. Both operations were well maintained. Extensive sampling of the swine barn and feedlot was conducted and continued in the swine barn once a month for the following five months.

### Laboratory investigation

*Escherichia coli* O157 was detected in 20/50 pork product samples collected from various premises as part of this investigation. *Escherichia coli* O157 was also detected in swine feces from the barn on the premises, in cattle feces from the feedlot on the premises, and a production area environmental swab (**Table 2**). Repeated swine barn sampling identified *E. coli* O157 each month in 7% to 15% of samples, indicating that the organism was persisting in the barn. A predominant PFGE cluster pattern was identified among clinical *E. coli* O157 outbreak isolates, which matched all food, environmental, swine and cattle fecal isolates. Isolates with this PFGE cluster pattern had not been seen in Alberta since 2017, when three isolates were identified.

**Table 2:** Food and environmental samples with *Escherichia coli* (*E. coli*) O157 detected, pork-associated outbreak of *E. coli* O157, Alberta, March–May 2018

Food facility	Sample results^a^ with *E. coli* O157 detected
Facility A	Raw pork parts^b^ (n=2), environmental samples: cattle feces (n=8), swine feces (n=4), environmental swab (n=1)
Restaurant A	Raw pork parts^b^ (n=2)
Retail Establishment A	Raw pork parts^b^ (n=1), raw sausage (n=1), ready-to-eat sausage containing raw pork (n=1)
Retail Establishment B	Raw pork parts^b^ (n=1), raw sausage^c^ (n=7)
Retail Establishment C	Raw ground pork^d^ (n=2)
Retail Establishment D	Raw pork parts^b^ (n=1)

Abbreviation: *E. coli*, *Escherichia coli*

^a^ Among 50 samples collected containing pork originating from Facility A, and 180 environmental samples collected at Facility A: environmental swab (n=128), swine feces (n=26), cattle feces (n=26)

^b^ Includes pork hock, leg, shoulder and/or chop

^c^ Collected from homes of outbreak cases (n=3) and from retail establishment (n=4)

^d^ Collected from home of outbreak case

The NML reported no other occurrences of the PFGE cluster pattern in the 60-day window prior to the outbreak. The wgMLST results indicate that the outbreak isolates (clinical, food and environmental) were closely related (a less than 10 allele difference among them) except one isolate (clinical) that differed by 12 alleles.

## Discussion

Between March 2018 to May 2018, an outbreak of 43 *E. coli* O157 infections in Alberta was identified through notifiable disease surveillance. The epidemiological, laboratory and food safety evidence collected during the investigation supported pork from a single meat facility as the primary source of this outbreak. The identified linkage was used to inform interventions aimed at limiting further illness transmission, including a recall of pork products from the implicated facility and improvements to its food handling processes. This investigation demonstrates a direct link between colonized swine, contaminated pork products, and human illness with *E. coli* O157.

At time of publication, this was the third significant pork-associated *E. coli* O157 outbreak in Alberta since 2014. Pork consumption patterns of Albertans would not seem to be a factor; weekly pork consumption frequency by Albertans (53.1%) is less than in most other Canadian provinces (10). Mishandling and/or undercooking of pork by several establishments in the distribution chain was a contributing factor for all three outbreaks. Consumer knowledge of the risks of consuming raw or undercooked pork may also have been a contributing factor, given that four outbreak cases consumed raw pork in a sausage sold as ready-to-eat, and one outbreak case consumed intentionally undercooked pork.

While *E. coli* O157 is considered endemic in beef cattle in Alberta, there has been limited research on its presence in swine in the province. A previous provincial abattoir survey did not detect *E. coli* O157 in pork carcasses (11), however, a more recent Alberta survey in 2017 (12) indicated that the bacteria is present in swine colon contents at a low level (1.4%). Control or eradication of infection in livestock has proven challenging and no effective practical methods have been developed.

In the same 2017 Alberta survey, *E. coli* O157 was not detected in pork carcass or swine colon content samples collected from Facility A, indicating a possible recent introduction to the swine barn. The presence of the organism in the cattle suggests the adjacent feedlot as a possible source. The swine barn had undergone an extensive renovation just prior to the outbreak, which may have increased the risk of exposure to *E. coli* O157 due to increased human traffic into the barn and reduced biosecurity during this period. However, since samples from both cattle and swine were taken during the same time period, no conclusion can be made about the cattle being infected first.

More than half of outbreak cases were epidemiologically linked to Restaurant A. This restaurant received pork from Facility A prior to and during the outbreak, but conditions and practices identified at the restaurant may also have contributed to transmission. Only seven of 12 restaurant patron cases reported pork consumption in their meals, which is suggestive of cross-contamination between raw pork received by the restaurant and other foods. This may have resulted from improper food handling practices observed during the investigation. For cases that reported consumption of food served in the “Boodle” style, the proximity of pork (if undercooked) to other foods served in this manner could result in cross-contamination. More than a third of employees were found to be infected with the *E. coli* O157 outbreak strain during the investigation, all but one of whom were reportedly asymptomatic. The period of communicability of the infected employees could not be confirmed, but it is plausible that they contributed to transmission.

## Limitations

A key limitation of this investigation was the inability to confirm the root cause of the colonization of swine and the contamination of pork products. Furthermore, this investigation and similar recent investigations in Alberta have not yielded information to determine why pork products are a recurring vehicle of transmission of human *E. coli* O157 in Alberta, and not in other jurisdictions in Canada.

## Conclusion

This outbreak investigation, combined with the two previous pork-associated *E. coli* O157 outbreaks in Alberta since 2014, provide evidence that pork should be considered to be a vehicle of transmission of *E. coli* O157 infections and should be included in consumer guidance for safe pork handling. The investigation findings also demonstrate that swine colonization with the pathogen may occur and contribute to risk of human illness, highlighting the importance of interventions during animal husbandry, biosecurity, slaughter, processing and handling to reduce the risk of *E. coli* O157 in pork products, especially when swine are raised in close proximity to beef cattle.
